# Diagnostic Laparoscopy as Decision Tool for Re-recurrent Inguinal Hernia Treatment Following Open Anterior and Laparo-Endoscopic Posterior Repair

**DOI:** 10.3389/fsurg.2017.00022

**Published:** 2017-05-01

**Authors:** Ferdinand Köckerling, Christine Schug-Pass

**Affiliations:** ^1^Department of Surgery, Center for Minimally Invasive Surgery, Academic Teaching Hospital of Charité Medical School, Vivantes Hospital, Berlin, Germany

**Keywords:** inguinal hernia, explorative laparoscopy, re-recurrence, guidelines, TAPP

## Abstract

**Introduction:**

The guidelines of the international hernia societies recommend posterior repair in laparo-endoscopic technique for recurrent inguinal hernia after open anterior mesh repair and, conversely, open anterior repair for recurrence after laparo-endoscopic primary repair. Even when these guidelines are followed, already 1 year after repair a re-recurrence rate of 1–2% must be expected, with that rate rising further in the subsequent years. Accordingly, increasingly more patients with re-recurrence after anterior and posterior mesh implantation must be treated, which constitutes a problem that to date has been investigated in only very few studies. Hence, there are no well-founded recommendations. This paper now presents a number of case reports aimed at identifying the role of explorative laparoscopy as decision tool for re-recurrent inguinal hernia treatment.

**Patients and methods:**

Based on three case reports the role of explorative laparoscopy as decision tool for re-recurrent inguinal hernia treatment is presented below.

**Results:**

In all the three cases described explorative laparoscopy played a key role as decision tool when deciding how best to treat re-recurrence after anterior and posterior inguinal hernia repair. In one case severe adhesions after robotic prostatectomy and in another case correct placement of the mesh in the posterior plane, adhesions from the cecum to the groin region and no definitive finding of a re-recurrence resulted in an open repair. In the third case, an insufficient laparoscopic posterior mesh placement made the re-recurrent TAPP procedure relatively easy.

**Conclusion:**

Explorative laparoscopy is an important decision tool for re-recurrent inguinal hernia treatment to minimize the risks of the procedure for the patients.

## Introduction

Operation for a recurrent inguinal hernia is common (12%) (1–4), and the risk of re-recurrence is high (5). In all guidelines of the international hernia societies, laparo-endoscopic recurrent inguinal hernia repair is recommended after failed open anterior tissue or Lichtenstein repair and open anterior repair in Lichtenstein technique after failed posterior laparo-endoscopic repair (6–11). Once an open anterior repair has been done, a laparo-endoscopic repair will generally go through nearly undisturbed tissue planes, permitting relative ease of dissection (11). After a failed TEP or TAPP repair, where the posterior extraperitoneal space was entered, it is strongly recommended that an open anterior mesh repair (Lichtenstein)—which does not involve entering the posterior space—should be performed (11).

After previous posterior laparo-endoscopic primary repair and open anterior recurrent operation, the re-recurrence rate on 1-year follow-up is 1.1% (4). Similarly, after posterior laparo-endoscopic recurrent operation following previous anterior open primary repair, a re-recurrence rate of 1.45% is seen on 1-year follow-up (4). Since the best results for recurrent inguinal hernia repair are obtained when following the guidelines (4), it is expected that in the future the recommendations for recurrent inguinal hernia repair will no doubt be implemented increasingly more often. If re-recurrence does present there is the problem that a mesh will have already been implanted into both the anterior and posterior tissue planes, which means that re-recurrent operation will present a technical challenge.

In such settings, the new “HerniaSurge Guidelines for groin hernia management” recommend that an expert surgeon should repair a re-recurrent inguinal hernia after failed anterior and posterior repair. The choice of technique depends on patient- and surgeon-specific factors (11). Clinical examination and ultrasound are according to the guidelines, the most suitable modalities for confirming the diagnosis of recurrent hernia (11). Dynamic MRI or CT can be considered for further evaluation if ultrasound is negative or non-diagnostic (11).

Laparoscopy represents a further diagnostic modality. Diagnostic laparoscopy can not only confirm once again the re-recurrence diagnosis but will provide further valuable information for better management of the re-recurrence operation. For example, this will help decide between a repeat of open or laparoscopic repair in the interest of risk minimization for the patient.

Based on three case reports, this paper now aims to demonstrate the advantages of diagnostic laparoscopy for management of re-recurrence after previous anterior and posterior inguinal hernia repair.

## Case Reports

### Patient 1

**Figure 1:**
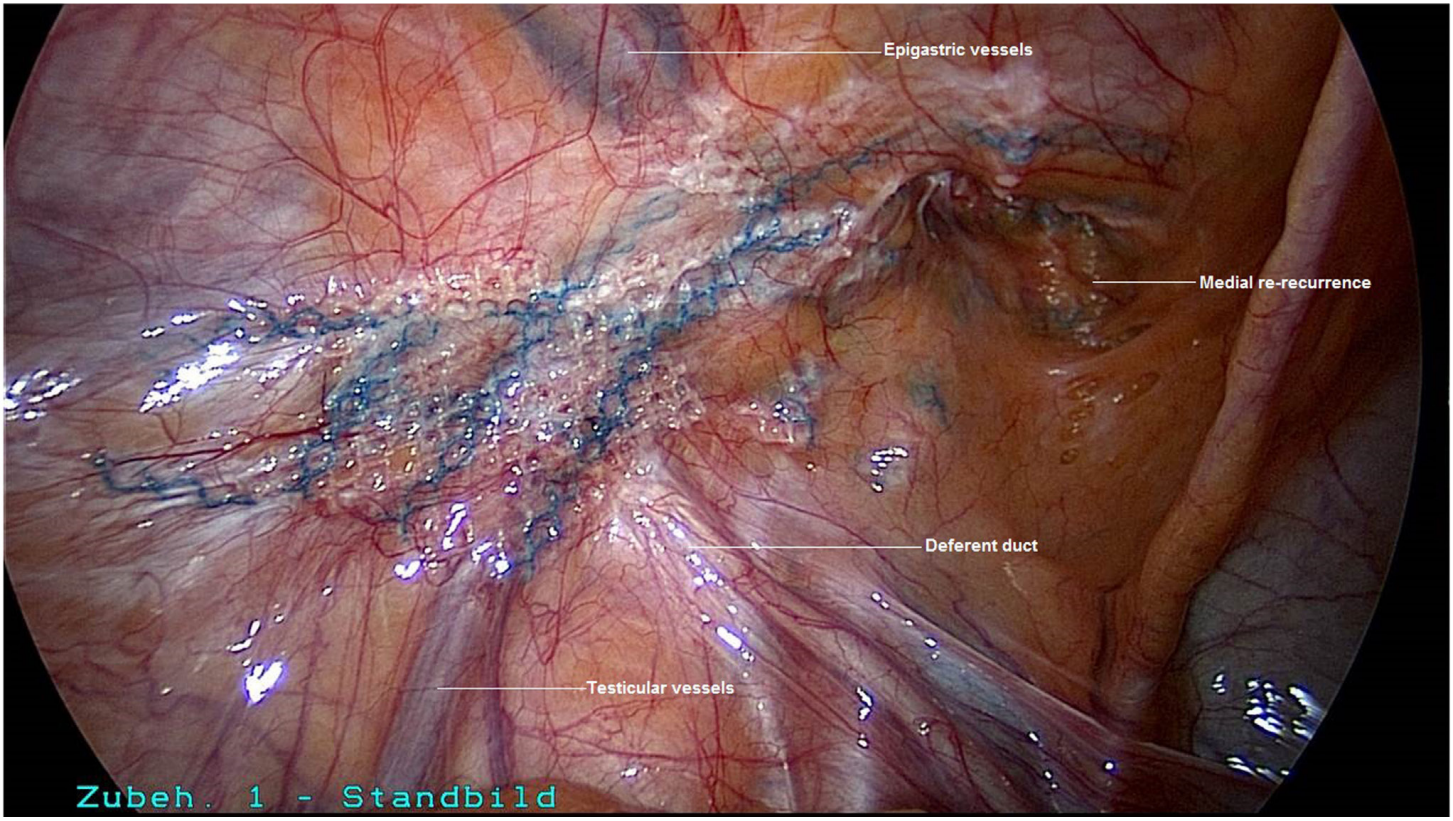
**Explorative laparoscopy of the left groin for re-recurrence following TAPP and Lichtenstein operation**.

**Figure 2:**
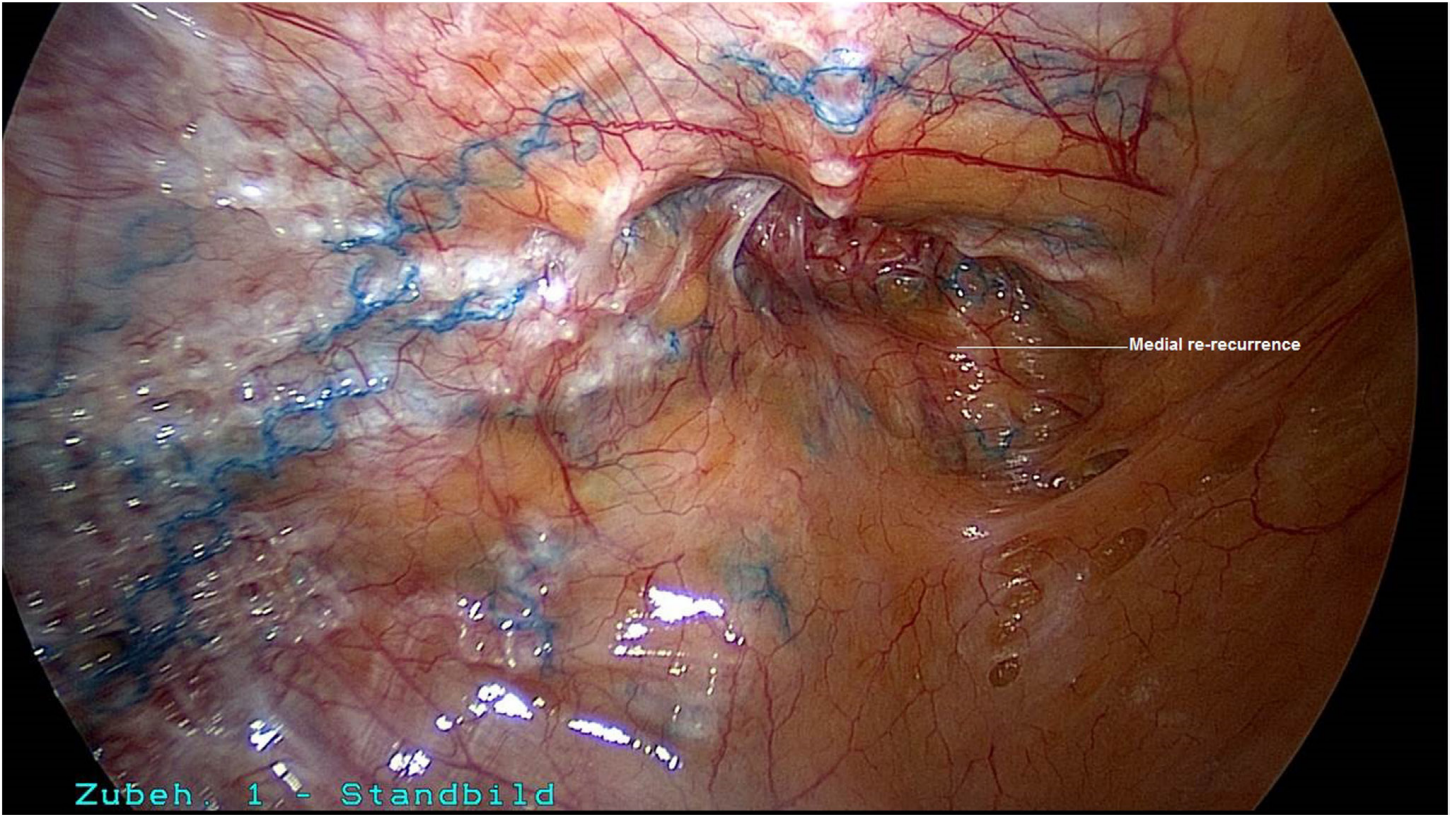
**Good visualization of the medial re-recurrence following TAPP and Lichtenstein operation (left groin)**.

**Figure 3:**
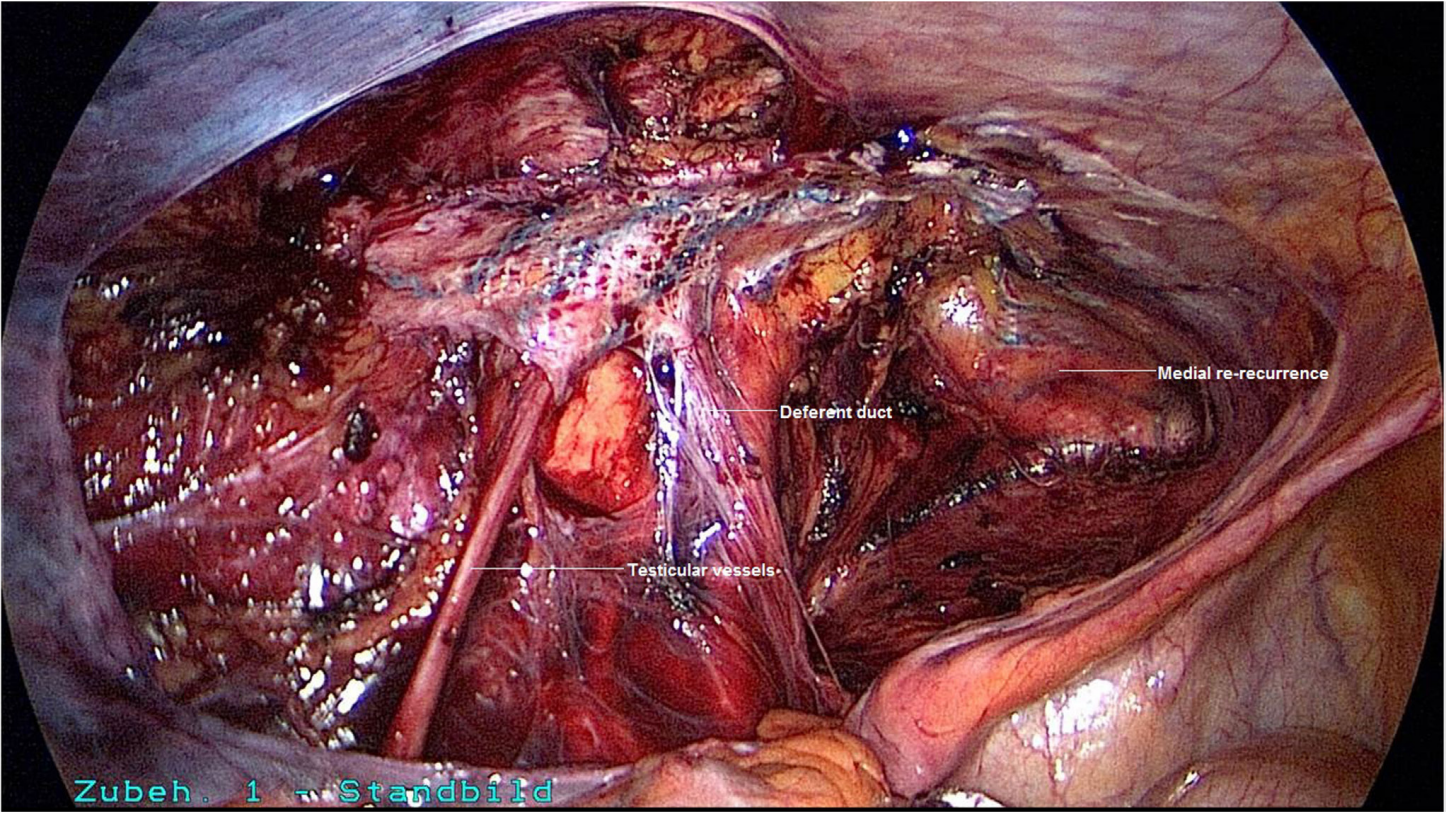
**Following incision of the peritoneum above and below the incorrectly positioned mesh, dissection of the left groin**.

**Figure 4:**
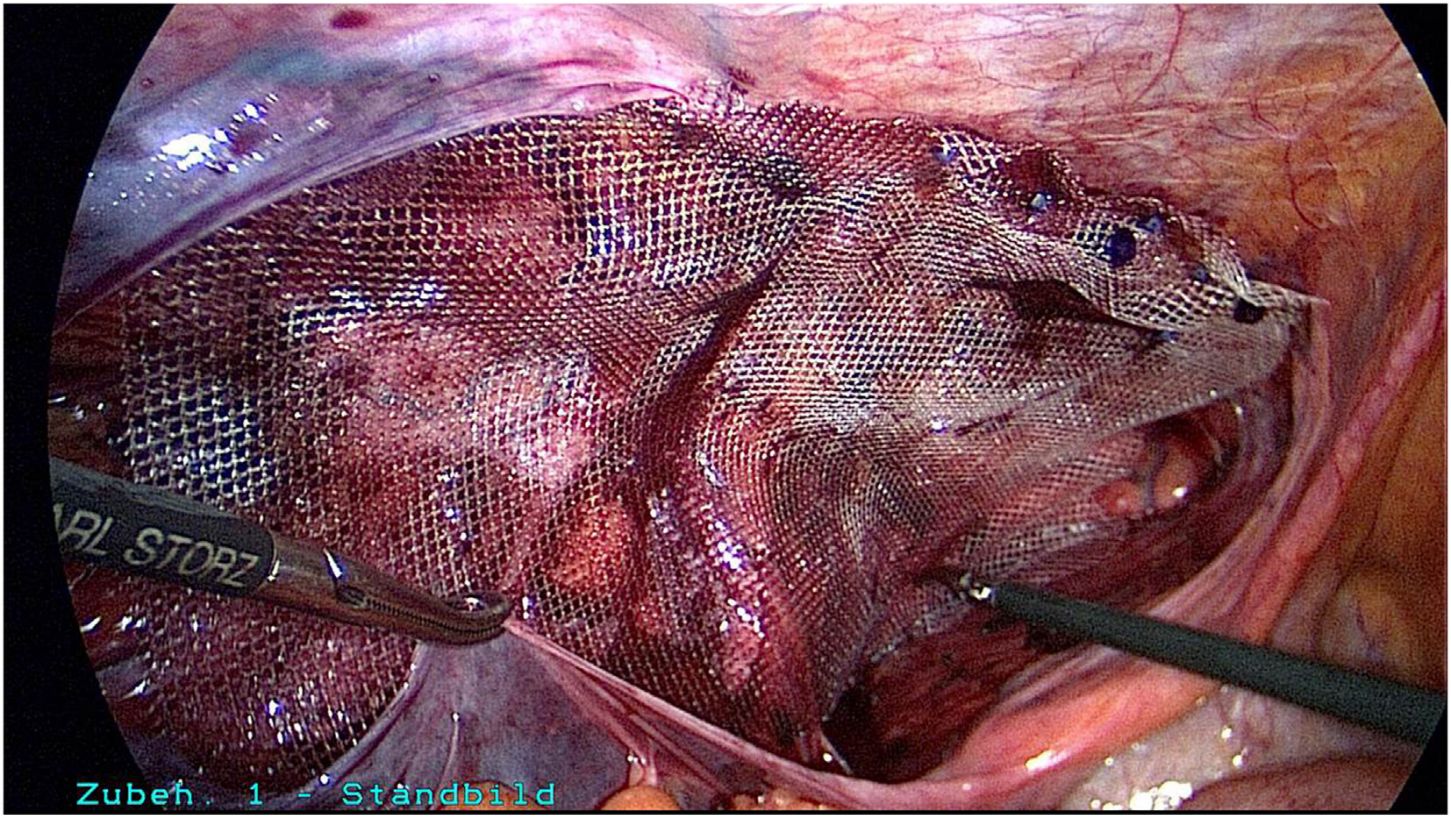
**Placement of a mesh measuring 10 cm × 15 cm (TiMesh light, pfm medical, Cologne) and fixation of the superior margin with absorbable tackers (SecureStrap, Ethicon, Norderstedt) (left groin)**.

**Figure 5:**
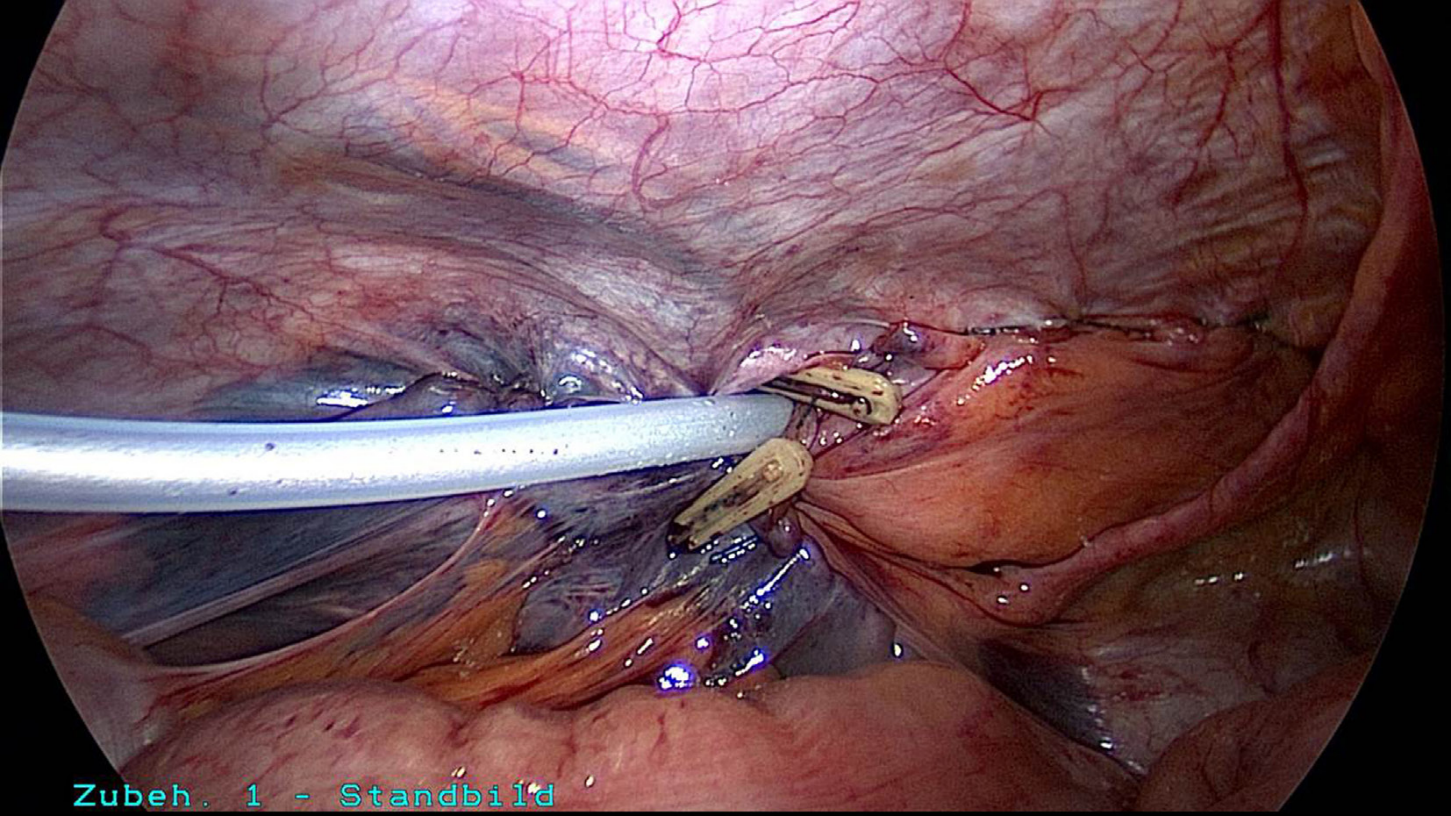
**Placement of a drain between the abdominal wall and peritoneum and closure of the peritoneal defect with a continuous suture (left groin)**.

A 50-year-old patient underwent TAPP operation in 2010 for primary unilateral inguinal hernia. This was followed in 2011 by Lichtenstein operation for recurrent repair. Then once again the patient experienced symptomatic recurrence. To confirm the diagnosis and assist in preoperative management we first carried out explorative laparoscopy, which revealed a medial re-recurrence with inadequate dissection and mesh placement at the time of primary repair in TAPP technique (Figures 1 and 2). Since we were easily able to perform dissection above and below the inadequately fitted mesh, re-TAPP was conducted (Figure 3). It was possible to fit a mesh measuring 15 × 10 cm (TiMesh light) on completion of dissection (Figure 4). In this case, the superior margin of the mesh was fixed with absorbable tackers (SecureStrap). After placement of a drain between the mesh and the peritoneum, the peritoneum was closed with a suture (Figure 5). The postoperative course was uneventful.

### Patient 2

**Figure 6:**
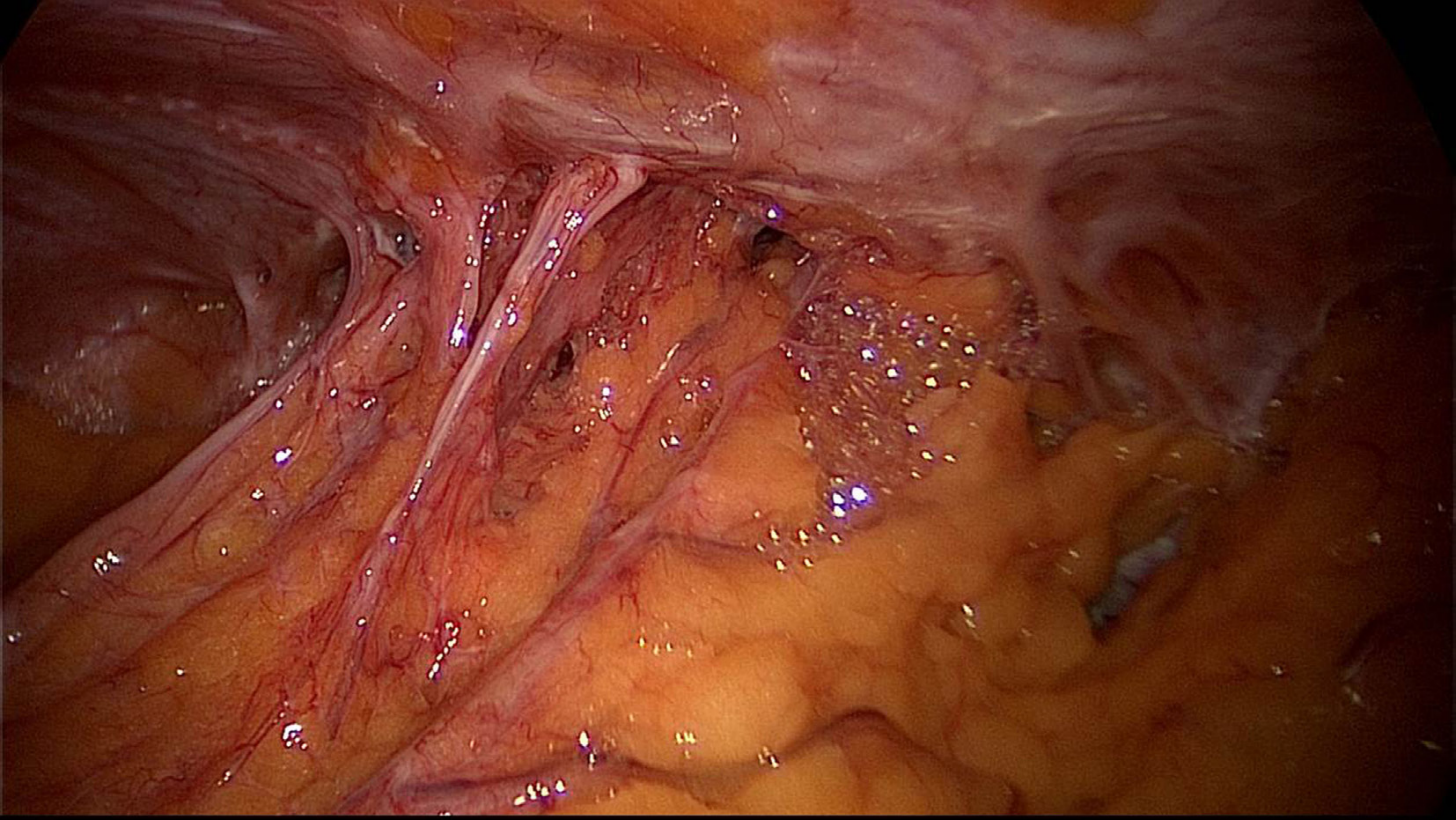
**Explorative laparoscopy of the left groin for re-recurrence following TAPP and Lichtenstein operation as well as robotic radical prostatectomy**.

**Figure 7:**
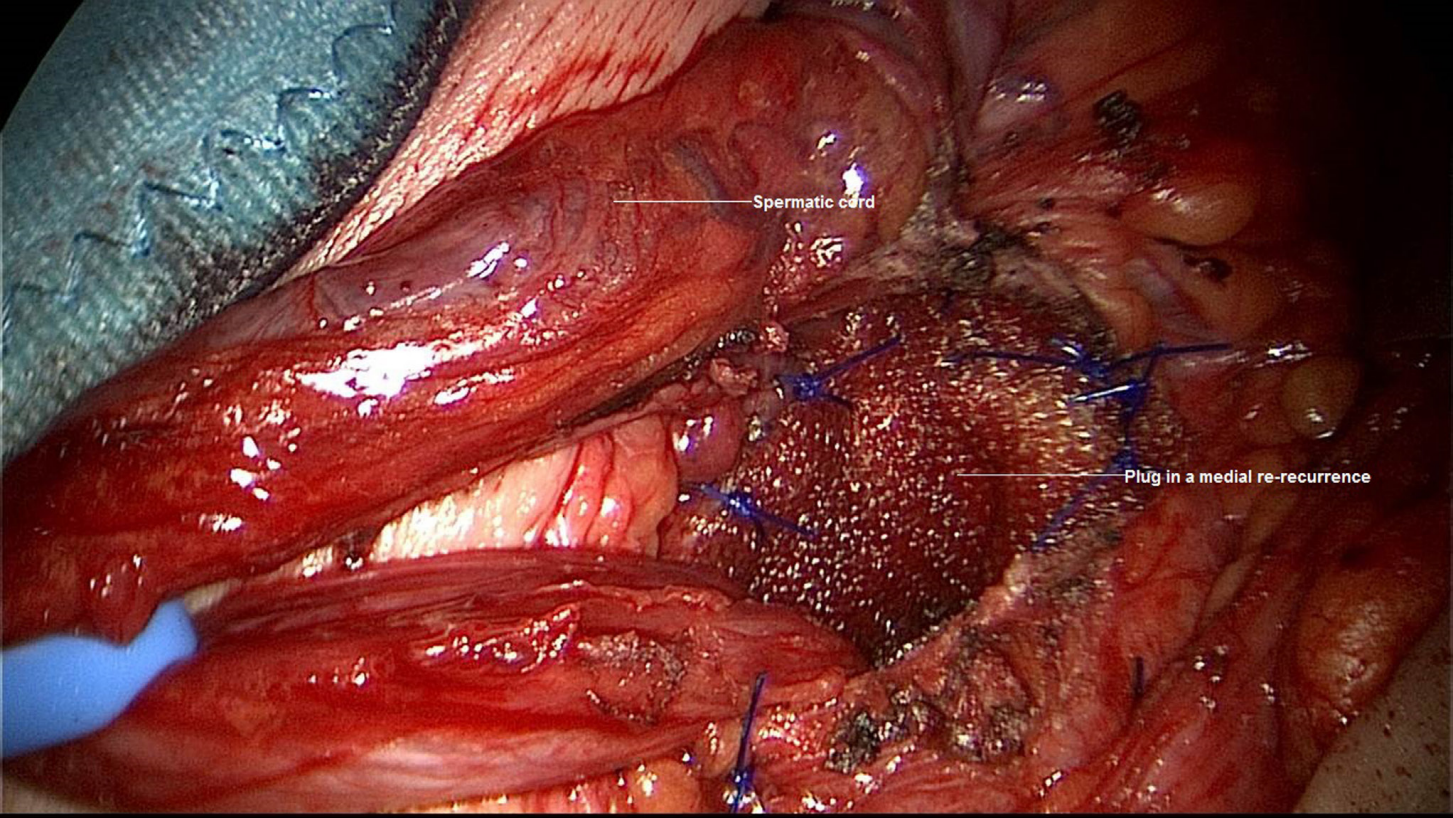
**Repair of medial re-recurrence after TAPP and Lichtenstein operation with a plug (left groin)**.

A 75-year-old patient underwent bilateral TAPP operation in 1998 for primary bilateral inguinal hernia. In March 2015, robotic radical prostatectomy had to be performed. In September 2016, the patient experienced symptomatic recurrent inguinal hernia, which was repaired in Lichtenstein technique. Around 1 year after Lichtenstein operation another recurrent inguinal hernia developed. We operated on that in January 2017 after first carrying out explorative laparoscopy. The latter revealed extensive adhesions in the region of the left groin (Figure 6) after radical prostatectomy, which ruled out repeat overview dissection of the left groin in laparoscopic technique. It was, therefore, decided to perform open operation once again. The medial recurrence was exposed and repaired in plug technique (Figure 7). The postoperative course was free of complications.

### Patient 3

**Figure 8:**
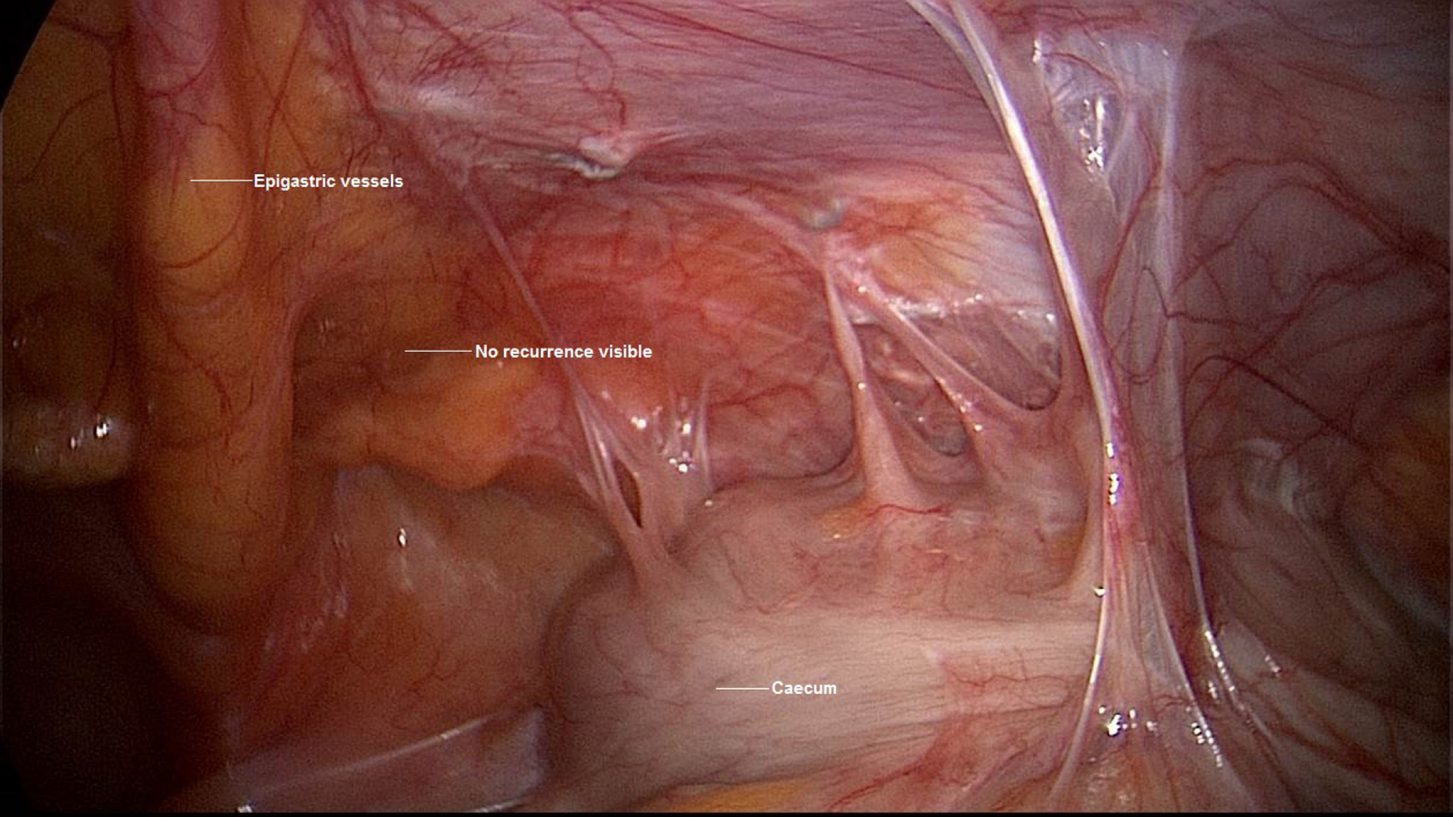
**Explorative laparoscopy for re-recurrence after TAPP and Shouldice operation (right groin)**.

**Figure 9:**
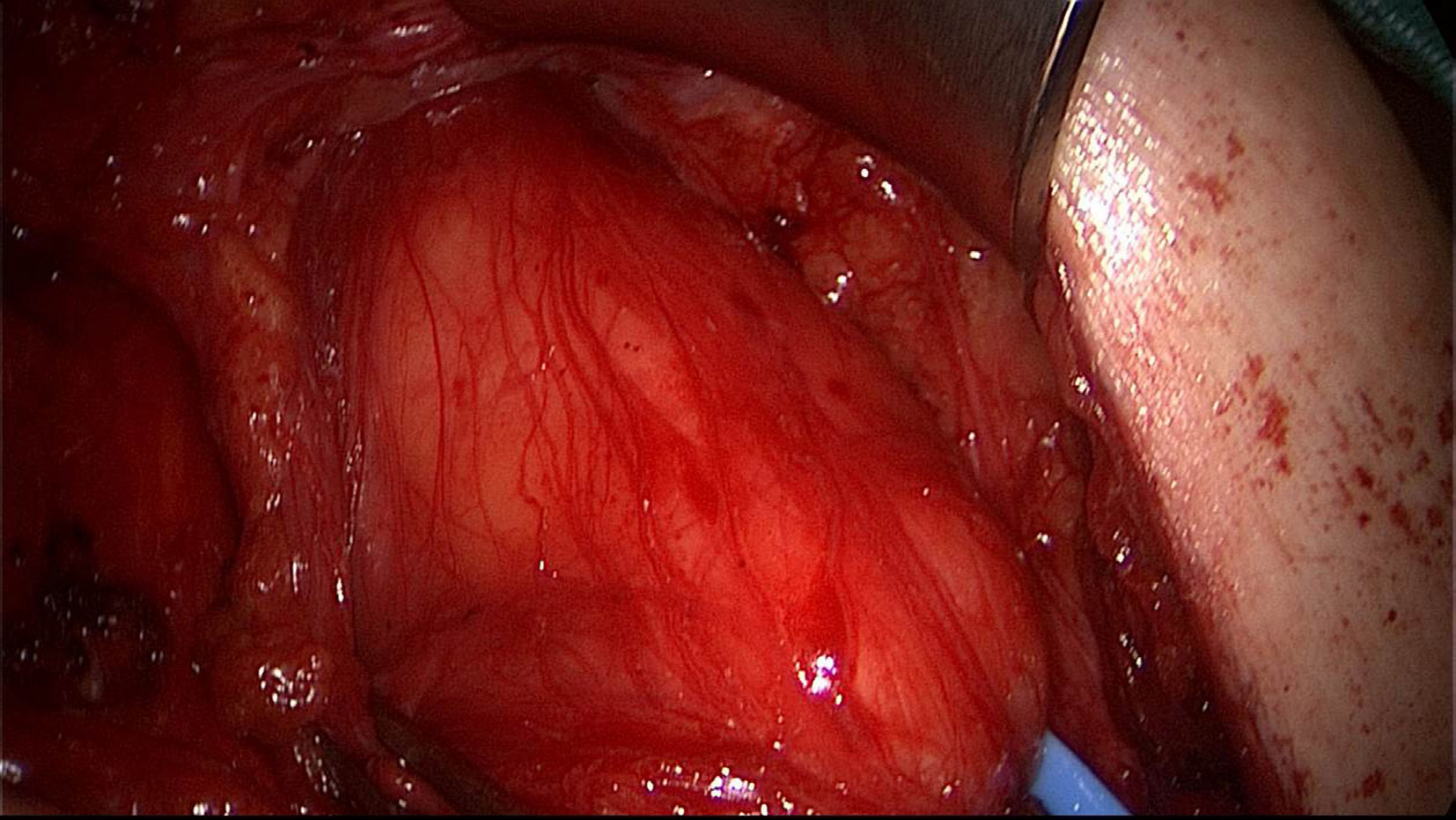
**Extensive lipoma and additional hernia sac identified during open re-recurrent operation (right groin)**.

**Figure 10:**
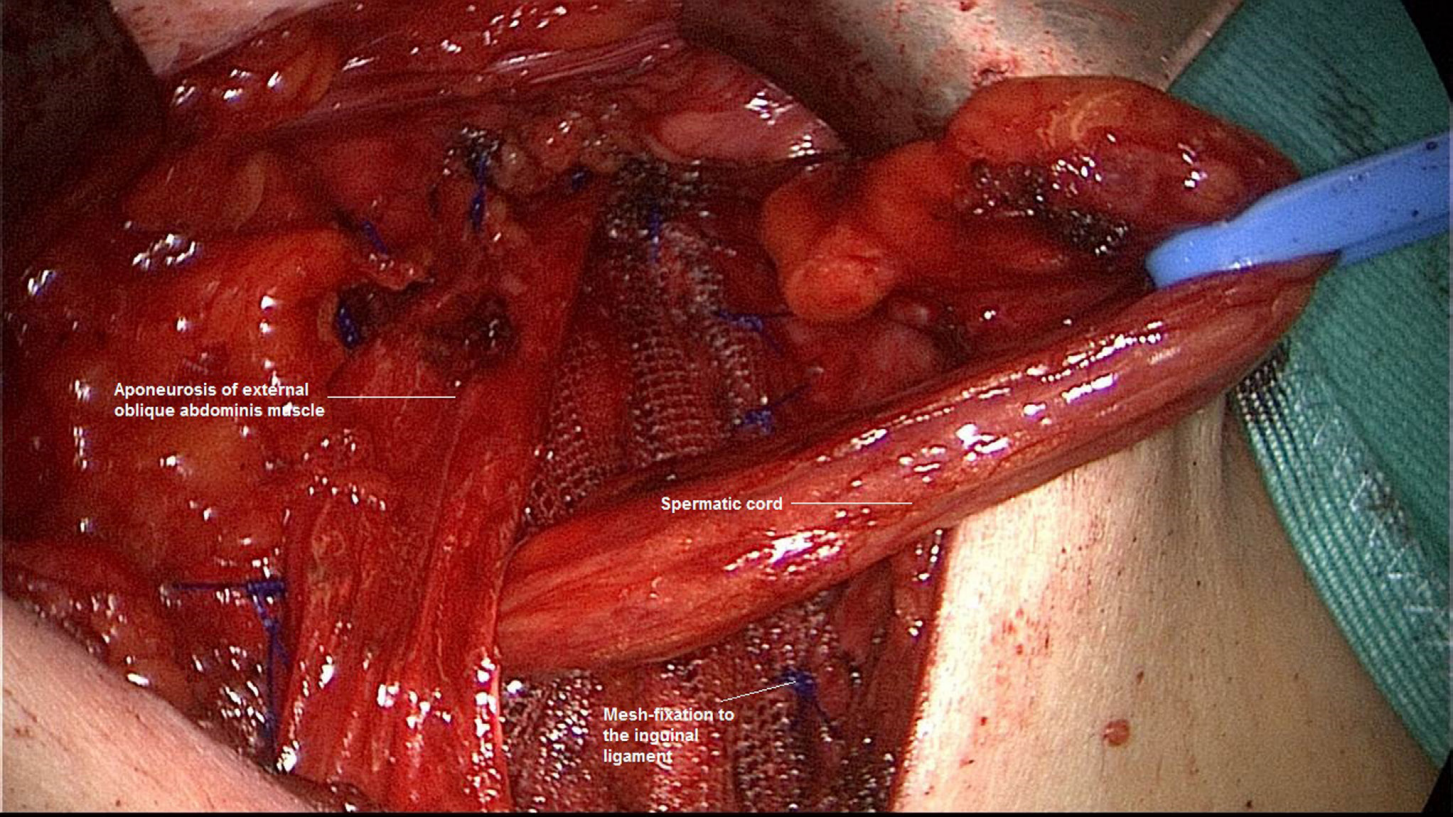
**Closure of the lateral re-recurrent hernia in Lichtenstein technique (right groin)**.

A 54-year-old patient underwent TAPP operation in 2006 for right primary inguinal hernia. This was followed in 2012 by Shouldice operation because of a recurrence. Then in 2016 the patient developed a symptomatic re-recurrence, which was treated by us again in January 2017. Explorative laparoscopy showed widespread adhesions of the cecum to the TAPP repair region (Figure 8). Otherwise, the mesh lay evenly in the groin. Without further dissection it was not possible to see the re-recurrence from the abdomen. It was decided to perform open Lichtenstein operation, which revealed a large lipoma in the region of the spermatic cord structures (Figure 9); this was excised and resected. Besides, a lateral hernia sac was identified and was also resected. Next Lichtenstein operation was carried out (Figure 10). The postoperative course was free of complications.

## Discussion

All guidelines of the international hernia societies recommend laparo-endoscopic repair for recurrence after primary open inguinal hernia repair and open repair for recurrence after laparo-endoscopic primary repair (6–11). While that approach assures the best outcome (4), even when these guidelines are followed a re-recurrence rate of 1–2% is seen on 1-year follow-up, with that rate rising further in the subsequent years (3). Accordingly, increasingly more patients will in the future seek treatment for re-recurrence after anterior and posterior mesh implantation in the groin. There is very little information in the literature on this topic (5). The new guidelines of the HerniaSurge group merely recommend that an expert surgeon should treat this patient and that the choice of technique should be based on patient- and surgeon-specific factors.

**Figure 11:**
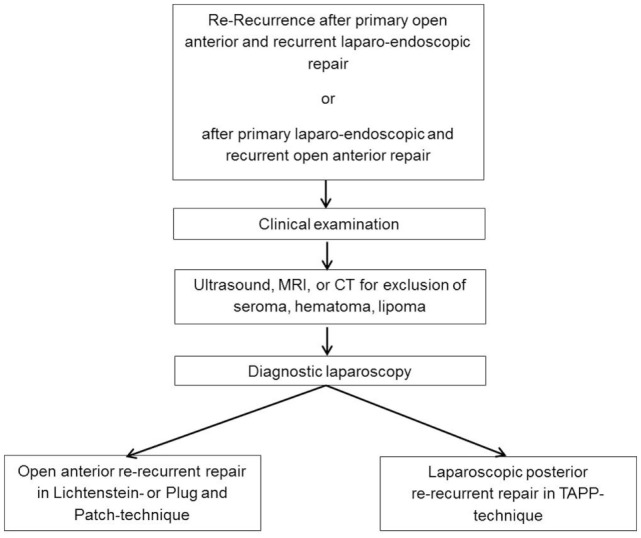
**Diagnostic and therapeutic algorithm in re-recurrent inguinal hernias after anterior and posterior primary and recurrent repair**.

The problem encountered when treating patients with re-recurrences following anterior and posterior mesh implantation is that a mesh has already been implanted into both anatomic tissue planes, which will hamper any further repair procedure. Diagnostic imaging modalities such as ultrasound, CT and MRI in general do not help decide which surgical technique should best be used, but can demonstrate findings like seroma, hematoma or lipoma. Based on our experience, explorative laparoscopy is a useful tool in this setting. As borne out by the cases described above, the explorative laparoscopy findings help decide which repair technique poses fewest risks to the patient and is likely to assure a better outcome (Figure 11). Armed with better insights, it can then be decided which repair technique is best. For example, this approach can reveal any drawbacks associated with previous posterior mesh implantation, and this might tend to favor a repeat posterior repair in laparoscopic technique. There is no difference in performing a laparoscopic re-recurrent procedure following a previous TAPP or TEP. The laparoscopic re-recurrent TAPP operation is the preferred technique after mesh placement in the preperitoneal space in the previous operation. On the other hand, extensive adhesions and changes to the groin region that would advise against laparo-endoscopic repair can be diagnosed. The aforementioned cases serve to show the advantages of explorative laparoscopy after previous anterior and posterior repair for treatment of re-recurrences. Based on our own experiences we can, therefore, recommend this diagnostic procedure, which is now a standard technique in our hospital. An important prerequisite for this approach is the expertise of the surgeon with laparo-endoscopic surgery that explorative laparoscopy and laparoscopic treatment of a re-recurrent inguinal hernia after previous posterior mesh placement can be performed with low complication risks for the patient. These demands represent the limitation of this approach.

In summary, it can be stated that diagnostic explorative laparoscopy is a useful decision tool for treatment of re-recurrence after anterior and posterior inguinal hernia repair.

## Ethics Statement

All subjects gave written informed consent in accordance with the Declaration of Helsinki.

## Author Contributions

FK: clinical cases, literature review, and manuscript writing. CS-P: literature review and manuscript writing.

## Conflict of Interest Statement

The authors declare that the research was conducted in the absence of any commercial or financial relationships that could be construed as a potential conflict of interest.
